# Millennium Development Goals in Vietnam: Taking Multi-sectoral Action to Improve Health and Address the Social Determinants

**DOI:** 10.3402/gha.v9.31271

**Published:** 2016-02-29

**Authors:** Hoang Van Minh, Juhwan Oh, Luu Ngoc Hoat, Jong-Koo Lee, Jennifer Stewart Williams

**Affiliations:** 1 Hanoi School of Public Health, Hanoi, Vietnam. Email: hvm@hsph.edu.vn; 2 Center for Global Medicine, Seoul National University College of Medicine, Seoul, Republic of Korea. Email: oh328@snu.ac.kr; 3 Hanoi Medical University, Hanoi, Vietnam; 4 Seoul National University College of Medicine, Seoul, South Korea; 5 Unit of Epidemiology and Global Health, Department of Public Health and Clinical Medicine, Faculty of Medicine, Umeå University, Umeå, Sweden

At the United Nations (UN) Millennium Summit held in New York in September 2000, all 189 UN member states committed to a new global partnership to reduce extreme poverty and to an agreed series of time-bound targets with a deadline of 2015. These targets, known as the Millennium Development Goals (MDGs) aim to: 1) eradicate extreme poverty and hunger; 2) achieve universal primary education; 3) promote gender equality and empower women; 4) reduce child mortality; 5) improve maternal health; 6) combat HIV/AIDS, malaria, and other diseases; 7) ensure environmental sustainability, and 8) develop a global partnership for development (1).

The Government of Vietnam has demonstrated strong political will in support of the MDGs by aligning many of its policies with these global targets. Moreover, as a way of taking ‘ownership’ of the MDGs, Vietnam has translated the MDGs into ‘actionable’ Vietnam Development Goals to better suit the specific political and social circumstances of the country. The Vietnam Development Goals are intended to meet the most basic needs of the Vietnamese population in terms of employment and income, health and education, water and sanitation. Yet inter-dependence between these sectors means that it is not effective to tackle any one sector in isolation. Accordingly, Vietnam has adopted a multi-sectoral approach to achieving positive sustainable change in progressing toward the MDGs.

The MDG framework places emphasis on the need to build a strong multi-sector scientific evidence base to inform policy and practice, and enable routine monitoring and evaluation of developments as they occur. The Government of Vietnam is strongly committed to building evidence on health and the social determinants (of health) for the health-related targets within the MDGs. This cluster of scientific papers is based on data from Vietnam's Multiple Indicator Cluster Surveys 2000, 2006, and 2011. Together with previous reports and research papers on the MDGs in Vietnam (2–6), the papers in this Special Issue of *Global Health Action* provide in-depth analyses of Vietnam's progress toward meeting specific health-related MDGs and addressing their social determinants.

In general, Vietnam has already achieved almost all the health-related MDGs (2–6). For MDG 3, Oanh et al. (7) found that the prevalence of acceptance of domestic violence has declined in Vietnam. Socioeconomic factors associated with women's condoning of domestic violence were age, wealth, educational level, and living area. Younger age and low educational attainment were key factors associated with violence-supportive attitudes, and these associations have become stronger over time.

One of the overarching goals of the MDGs is to reduce infant and under-five childhood mortality. A paper by Lee et al. (8) confirmed that Vietnam has already achieved MDG 4. The infant and under-five mortality rates significantly decreased for both male and female infants and children between 1986 and 2011. However the paper also illustrates inequity problems in child mortality in Vietnam. Women who were living in the northern midlands Northern Midlands and Mountainous areas were more likely to experience child deaths. Women who were from minor ethnic groups, had low education, were living in urban areas, and had multiple children were more likely to have experienced child deaths. Another paper by Lee et al. (9) reported that mothers with higher levels of education had higher odds of seeking oral rehydration therapy for children with diarrhea, compared with mothers with the lowest level of education. Seeking formal treatment for children who have an illness with a cough was associated with being in a household in the richest wealth quintile. In the same line, Kien et al. (10) showed that the rate of under-five child malnutrition in Vietnam has significantly decreased. However, there were still significant differences in under-five child malnutrition that favored those who were more advantaged in socioeconomic terms. The impact of socioeconomic inequality on inequalities in child malnutrition has increased in Vietnam in recent years.

Immunization has been shown to be the single most important public health intervention which spares millions of children from diseases. The coverage of immunization has always been high in Vietnam (2–6). However Dao et al. (11) revealed that the proportion of children under five who had timely immunization completion was low, especially for HBV dose 2 and HBV dose 3, both of which decreased between 2000 and 2011. Timely immunization completion was less common among children whose mothers had relatively less household wealth, were from ethnic minorities, lived in rural areas, and had less education. Early initiation of breastfeeding followed by exclusive breastfeeding can improve the health and survival status of newborns but Bui et al. (12) identified a decreasing trend in both types of breastfeeding in Vietnam. Apart from child's age, individual-level characteristics were not significant predictors of breastfeeding, but there were strong associations with provincial characteristics.

Regarding progress toward meeting MDG 5, Minh et al. (13) found that there were increases in the proportion of women who received antenatal care by skilled staff and also in the proportion of women who gave birth with assistance from skilled staff. However the receipt of antenatal care by skilled staff and birth assistance from skilled health personnel was much less common in socially and economically vulnerable women, especially among those with multiple vulnerabilities. Vu et al. (14) revealed that individual factors, such as women's age, the number of living children, and having a son, were significantly associated with the use of modern contraceptives, however the provincial poverty rate mediated association between the individual's socioeconomic status and modern contraceptive use. The authors showed that the likelihood of married women using modern contraceptives varied significantly across Vietnam's 63 provinces. A counter-intuitive finding was that women of lower socioeconomic status in poorer provinces were more likely to use modern contraceptives.

Vietnam has also made significant improvements in the legal and policy framework related to MGD 6 (2–6). However Nguyen et al. (15) found identified a low prevalence of women in the general population with basic HIV knowledge and positive attitudes toward HIV. Women who have higher levels of education, live in urban areas, have higher socioeconomic status, and know about places for HIV-related services, were more likely to have good knowledge about HIV. Son et al. (16) reported that early sexual intercourse was significantly associated with having lifetime multiple sexual partners. There was significant association with having multiple sexual partners, for women with lower household wealth and of urban residence but the association with educational attainment was not strong.

Tuyet-Hanh et al. (17) investigated issues related to MDG 7 and found some outstanding developments in access to improved water sources and sanitation facilities in Vietnam, meeting the 2015 MDG targets for safe drinking water and basic sanitation. However households in urban compared with rural areas were more than twice as likely to have access to improved water and sanitation facilities in combination, and households in the highest compared with the lowest wealth quintile were over 40 times more likely. To et al. (18) showed a high prevalence of hand washing in Vietnam. Nevertheless, individuals with low education, low household wealth, belonging to ethnic minority groups, and with limited access to improved sanitation facilities and water sources, had poor hand washing practices.

A paper by Kim et al. (19) reported some improvements in birth registration, preschool education attendance, and parental support for early childhood education, however the possession of books was low. Inequalities in the ownership of books, as well as in the care and education of children, have persisted over time. The largest inequalities were for household wealth and rural versus urban residence.

In summary, this series of papers reveals that Vietnam has achieved outstanding progress in moving towards meeting the MDGs. Yet Vietnam has not fully overcome all the obstacles in the path of ensuring equitable health developments. Inequalities and inequities in health resulting from differential impacts of the social determinants on geographic regions and between socioeconomic groups present ongoing major challenges. Some of the remaining tasks are clearly identified in this series of papers (2–19). In the post-2015 period, the era of the Sustainable Development Goals, Vietnam will need to maintain continuing efforts to meet the targets and achieve equity in the distribution of health and access to affordable care.

Policy efforts in Vietnam should be directed toward closing the gaps in health between different socioeconomic groups. In other words, inequities in health and the social determinants must be more efficiently and effectively addressed. Policy makers, clinicians, researchers, scientists, and health staff operating at all levels need to work together using available resources to ensure that this is achieved in an expedient manner. We believe that the scientific evidence from this cluster of papers offers a valuable building block for policy formulation, development and intervention in Vietnam and elsewhere. We trust that these efforts will encourage other low- and middle-income countries to undertake similar research with regard to MDG achievements and challenges in their countries.

*Hoang Van Minh* Hanoi School of Public Health Hanoi, Vietnam Email: hvm@hsph.edu.vn *Juhwan Oh* Center for Global Medicine Seoul National University College of Medicine Seoul, Republic of Korea Email: oh328@snu.ac.kr *
Luu Ngoc Hoat* Hanoi Medical University Hanoi, Vietnam *Jong-Koo Lee* Seoul National University College of Medicine Seoul, South Korea *Jennifer Stewart Williams* Unit of Epidemiology and Global Health Department of Public Health and Clinical Medicine Faculty of Medicine, Umeå University Umeå, Sweden
